# Multiscale Modeling Framework of Ventricular-Arterial Bi-directional Interactions in the Cardiopulmonary Circulation

**DOI:** 10.3389/fphys.2020.00002

**Published:** 2020-01-31

**Authors:** Sheikh Mohammad Shavik, Christopher Tossas-Betancourt, C. Alberto Figueroa, Seungik Baek, Lik Chuan Lee

**Affiliations:** ^1^Department of Mechanical Engineering, Michigan State University, East Lansing, MI, United States; ^2^Department of Mechanical Engineering, Bangladesh University of Engineering and Technology, Dhaka, Bangladesh; ^3^Department of Biomedical Engineering, University of Michigan, Ann Arbor, MI, United States; ^4^Department of Surgery, University of Michigan, Ann Arbor, MI, United States

**Keywords:** pulmonary arterial hypertension (PAH), cardiac mechanics, vascular mechanics, image-based modeling, ventricular-arterial coupling

## Abstract

Ventricular-arterial coupling plays a key role in the physiologic function of the cardiovascular system. We have previously described a hybrid lumped-finite element (FE) modeling framework of the systemic circulation that couples idealized FE models of the aorta and the left ventricle (LV). Here, we describe an extension of the lumped-FE modeling framework that couples patient-specific FE models of the left and right ventricles, aorta and the large pulmonary arteries in both the systemic and pulmonary circulations. Geometries of the FE models were reconstructed from magnetic resonance (MR) images acquired in a pediatric patient diagnosed with pulmonary arterial hypertension (PAH). The modeling framework was calibrated with pressure waveforms acquired in the heart and arteries by catheterization as well as ventricular volume and arterial diameter waveforms measured from MR images. The calibrated model hemodynamic results match well with the clinically-measured waveforms (volume and pressure) in the LV and right ventricle (RV) as well as with the clinically-measured waveforms (pressure and diameter) in the aorta and main pulmonary artery. The calibrated framework was then used to simulate three cases, namely, (1) an increase in collagen in the large pulmonary arteries, (2) a decrease in RV contractility, and (3) an increase in the total pulmonary arterial resistance, all characteristics of progressive PAH. The key finding from these simulations is that hemodynamics of the pulmonary vasculature and RV wall stress are more sensitive to vasoconstriction with a 10% of reduction in the lumen diameter of the distal vessels than a 67% increase in the proximal vessel's collagen mass.

## Introduction

Ventricular-arterial coupling plays a vital role in the physiologic function of the cardiopulmonary circulation as well as in the evolution of cardiovascular diseases, such as pulmonary arterial hypertension (PAH) (Borlaug and Kass, 2011; Ky et al., 2013). In physiologic conditions, the arterial compliance (endowed by arterial wall tissue constituents) and the ventricular dynamic stiffness (inherent from the contraction of myocytes) confine the dynamic pressure variation to a physiological range to prevent end organ damage, while providing sufficient blood flow to meet oxygen demand of the body under varying workload (Borlaug and Kass, 2011). In pathological conditions, such as PAH, malfunction of one compartment (e.g., microcirculation) in the cardiopulmonary circulation may affect other compartments (e.g., ventricle) through a positive feedback loop that is driven by the tight coupling of ventricular and arterial systems, ultimately leading to end-stage heart failure. A modeling framework that captures the complex ventricular-arterial coupling would help elucidate the mechanisms governing the progression of PAH.

Existing mathematical modeling frameworks describing ventricular-arterial coupling in the cardiopulmonary circulation can be broadly classified as either a lumped parameter or a multi-scale finite element (FE) modeling framework. In a lumped parameter modeling framework, the ventricular-arterial coupling is described by an electrical analog representation of the cardiovascular system (Ursino, 1998; Smith et al., 2004). While such modeling framework is computationally inexpensive, it cannot directly take into account detailed geometrical and microstructural features associated with pathological conditions in the ventricles and arteries. In a hybrid lumped-FE modeling framework, a FE model describing either ventricular mechanics (Kerckhoffs et al., 2007; Shavik et al., 2017, 2019) or arterial hemodynamics (Lau and Figueroa, 2015; Zambrano et al., 2018) is coupled to lumped-parameter representation of the other compartments to provide a detailed description of the cardiovascular system. To overcome limitations associated with simplified representations of cardiovascular components, we previously introduced a hybrid lumped-FE modeling framework that bidirectionally couples FE models of the aorta and left ventricle (LV) mechanics in a closed-loop circulatory system (Shavik et al., 2018). Based on an idealized geometry of the LV and aorta, the modeling framework is able to reproduce pressure, arterial diameter, and LV volume waveforms found in a healthy individual. The modeling framework, however, considers only the systemic circulation and does not take into account the pulmonary circulation.

Here, we describe the extension of our earlier framework (Shavik et al., 2018) in which image-based FE models of the large pulmonary arteries, aorta, and heart (including both ventricles) are coupled bidirectionally in a closed-loop multi-scale FE modeling framework of the cardiopulmonary circulation. The multi-scale framework was calibrated using *in vivo* clinical measurements of the anatomy, deformation, and hemodynamics from a PAH pediatric patient. Using the calibrated model, we further investigate how changes associated with the mechanical behavior and microstructure of the microcirculation, large pulmonary arteries, and right ventricle (RV), consequent of PAH progression, affect each other.

## Methods

This study was approved by the University of Michigan Board of Review (HUM00117706), and informed consent was obtained from the parents/guardians of the patient.

### Patient History

Clinical data was prospectively acquired in a 11-year-old female patient who was diagnosed with PAH. The patient had an elevated mean pulmonary arterial pressure (mPAP) of 59 mmHg with normal pulmonary capillary wedge pressure (PCWP) of 6 mmHg and elevated pulmonary vascular resistance (PVR) of 13.5 WU, falling within the clinical classification of PAH (mPAP ≥ 20 mmHg, PCWP ≤ 15 mmHg, and PVR ≥ 3 WU) (Simonneau et al., 2019). She has family history of chronic obstructive pulmonary disease and PAH.

### Data Acquisition

Anatomical and hemodynamic data were obtained using magnetic resonance (MR) imaging and arterial catheterization. Cine MR images of the short- and long-axis views of the ventricles were acquired at 30 time points in the cardiac cycle. Using the cine MR images, left and right ventricular endocardial surfaces were segmented with the medical image analysis software MeVisLab (www.mevislab.de) to acquire ventricular volume waveforms. Cardiac-gated gradient echo MR images of the vascular anatomy were acquired in the diastolic phase. Luminal area waveforms were also acquired with phase-contrast MR images (PC-MRI) at the ascending aorta and main pulmonary artery. Arterial catheterization was performed to acquire pressure waveforms in the LV, RV, main pulmonary artery (MPA), and aorta. The ventricular volume and pressure waveforms were synchronized to reconstruct pressure-volume (PV) loops (Xi et al., 2016; Shavik et al., 2019). Hemodynamic and cardiovascular function metrics of the PAH patient are listed in Table 1.

**Table 1:** Hemodynamic measurements of PAH patient.

**Quantities**	**Values**
HR, bpm	75
LVEDV, ml	72
LVESV, ml	25
LVEF, %	65
MAP, mmHg	68
RVEDV, ml	77
RVESV, ml	30
RVEF, %	61
RVEDV/LVEDV	1.07
mPAP, mmHg	59
PCWP, mmHg	6

### Biventricular and Vascular Geometries

**Figure 1:**
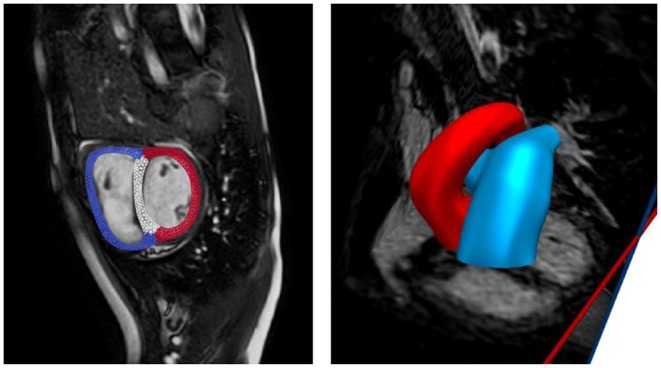
Reconstruction of biventricular model **(left)** and large proximal arteries **(right)** from cine MR images.

Anatomical models of the LV, RV, aorta, and pulmonary arteries (PA) (consisting of the main, left, and right pulmonary arteries) were reconstructed from the acquired MR images. The biventricular model was reconstructed from images that correspond to the point in the cardiac cycle where ventricular pressures were lowest during filling (Geuzaine and Remacle, 2009). Furthermore, anatomical models of the aorta and large pulmonary arteries were reconstructed using the blood flow modeling software CRIMSON (www.crimson.software) (Figure 1).

### Closed Loop Circulatory System

**Figure 2:**
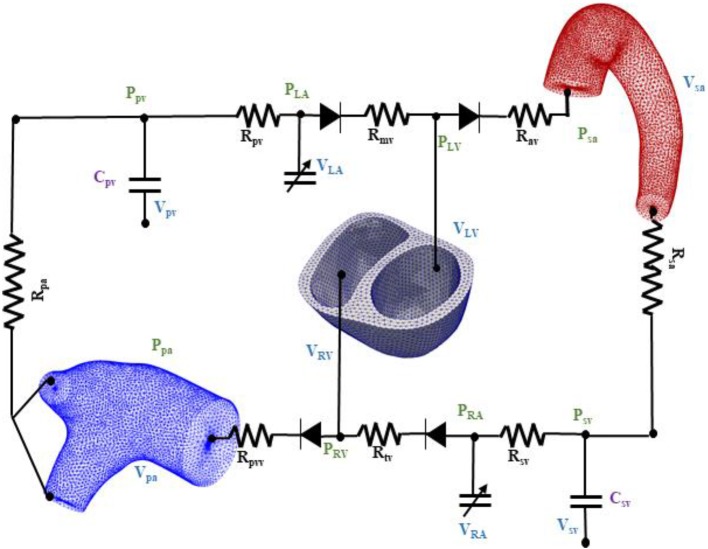
Schematic of the finite element ventricular-vascular coupling closed loop circulatory modeling framework.

The biventricular, aorta and pulmonary artery FE models were coupled through a closed loop lumped-parameter circulatory model that describes both systemic and pulmonary circulations (Figure 2). The modeling framework consists of eight compartments with four cardiovascular components (ventricle, atrium, artery, and vein) each in the systemic and pulmonary circulations. Conservation of total blood mass in the circulatory model requires the net change of inflow and outflow rates of each compartment to be related to the rate of change of the volume by the following relations

(1a) $$\begin{array}{l} \frac{dV_{LV}\left(t\right)}{dt}=q_{mv}\left(t\right)-q_{av}\left(t\right), \end{array}$$

(1b) $$\begin{array}{l} \frac{dV_{sa}\left(t\right)}{dt}=q_{av}\left(t\right)-q_{sa}\left(t\right), \end{array}$$

(1c) $$\begin{array}{l} \frac{dV_{sv}\left(t\right)}{dt}=q_{sa}\left(t\right)-q_{sv}\left(t\right), \end{array}$$

(1d) $$\begin{array}{l} \frac{dV_{RA}\left(t\right)}{dt}=q_{sv}\;\left(t\right)-q_{tv}\left(t\right), \end{array}$$

(1e) $$\begin{array}{l} \frac{dV_{RV}\left(t\right)}{dt}=q_{tv}\left(t\right)-q_{pvv}\left(t\right), \end{array}$$

(1f) $$\begin{array}{l} \frac{dV_{pa}\left(t\right)}{dt}=q_{pvv}\left(t\right)-q_{pa}\left(t\right) \end{array}$$

(1g) $$\begin{array}{l} \frac{dV_{pv}\left(t\right)}{dt}=q_{pa}\left(t\right)-q_{pv}\left(t\right), \end{array}$$

(1h) $$\begin{array}{l} \frac{dV_{LA}\left(t\right)}{dt}=q_{pv}\;\left(t\right)-q_{mv}\left(t\right). \end{array}$$

In Equation (1), *V*_*LV*_, *V*_*sa*_, *V*_*sv*_, *V*_*RA*_, *V*_*RV*_, *V*_*pa*_, *V*_*pv*_, and *V*_*LA*_ are the volumes of the eight compartments with the subscripts denoting the LV, systemic arteries (sa), systemic veins (sv), right atrium (RA), RV, pulmonary arteries (pa), pulmonary veins (pv), and left atrium (LA), respectively. Flow rates at different segments of the circulatory model are denoted by *q*_*mv*_, *q*_*av*_, *q*_*sa*_, *q*_*sv*_, *q*_*tv*_, *q*_*pvv*_, *q*_*pa*_, and *q*_*pv*_.

Systemic and pulmonary arteries and veins were modeled using their electrical analogs based on Ohm's law. At each segment, the flow rate depends on the pressure gradient and resistance to the flow as described in the following equation

(2a) $$\begin{array}{l} q_{mv}(t)=\{\begin{array}{ll} \frac{P_{LA}(t)\;-\;P_{LV}(t)}{R_{mv}} & when,\;P_{LA}(t)\geq P_{LV}(t)\\ 0 & when,\;P_{LA}(t)<P_{LV}(t) \end{array} \end{array}$$

(2b) $$\begin{array}{l} q_{av}(t)=\{\begin{array}{ll} \frac{P_{LV}(t)\;-\;P_{sa}(t)}{R_{av}} & when,\;P_{LV}(t)\geq P_{sa}(t)\\ 0 & when,\;P_{LV}(t)<P_{sa}(t) \end{array}, \end{array}$$

(2c) $$\begin{array}{l} q_{sa}\left(t\right)=\frac{P_{sa}\left(t\right)-P_{sv}\left(t\right)}{R_{sa}}, \end{array}$$

(2d) $$\begin{array}{l} q_{sv}\left(t\right)=\frac{P_{sv}\left(t\right)-P_{RA}\left(t\right)}{R_{sv}}\;, \end{array}$$

(2e) $$\begin{array}{l} q_{tv}(t)=\{\begin{array}{ll} \frac{P_{RA}(t)-P_{RV}(t)}{R_{tv}} & when,\;P_{RA}(t)\geq P_{RV}(t)\\ 0 & when,\;P_{RA}(t)<P_{RV}(t) \end{array}, \end{array}$$

(2f) $$\begin{array}{l} q_{pvv}(t)=\{\begin{array}{ll} \frac{P_{RV}(t)\;-\;P_{pa}(t)}{R_{pvv}} & when,\;P_{RV}(t)\geq P_{pa}(t)\\ 0 & when,\;P_{RV}(t)<P_{pa}(t) \end{array}, \end{array}$$

(2g) $$\begin{array}{l} q_{pa}\left(t\right)=\frac{P_{pa}\left(t\right)-P_{pv}\left(t\right)}{R_{pa}}, \end{array}$$

(2h) $$\begin{array}{l} q_{pv}\left(t\right)=\frac{P_{pv}\left(t\right)-P_{LA}\left(t\right)}{R_{pv}}. \end{array}$$

In Equation (2), *R*_*mv*_, *R*_*av*_, *R*_*tv*_, and *R*_*pvv*_ are the resistances associated with the mitral, aortic, tricuspid, and pulmonary valves, respectively. The valves are each represented by a diode that only permits one-way flow as in previous studies (Punnoose et al., 2012; Shavik et al., 2019). The vessel resistances are denoted by *R*_*sa*_, *R*_*sv*_, *R*_*pa*_, and *R*_*pv*_, respectively. To describe the compliance of the systemic and pulmonary vessels, we used the following PV relationships

(3a) $$\begin{array}{l} P_{sv}\left(t\right)=\frac{V_{sv}\left(t\right)-V_{sv,0}}{C_{sv}}, \end{array}$$

(3b) $$\begin{array}{l} P_{pv}\left(t\right)=\frac{V_{pv}\left(t\right)-V_{pv,0}}{C_{pv}}, \end{array}$$

where *V*_*sv*,0_ and *V*_*pv*,0_ are the resting volumes and *C*_*sv*_ and *C*_*pv*_ are the total compliance of the systemic and pulmonary veins, respectively.

Contraction of the LA and RA was modeled using a time varying elastance function that is given by the following PV relations

(4a) $$\begin{array}{l} P_{k}\left(t\right)=e\left(t\right)P_{es,k}\left(V_{k}\left(t\right)\right)+\left(1-e\left(t\right)\right)\;P_{ed,k}\left(V_{k}\left(t\right)\right), \end{array}$$

where,

(4b) $$\begin{array}{l} P_{es,k}\left(V_{k}\left(t\right)\right)=E_{es,k}\left(V_{k}\left(t\right)-V_{0,k}\right), \end{array}$$

(4c) $$\begin{array}{l} P_{ed,k}\left(V_{k}\left(t\right)\right)=A_{k}\;\left(e^{B_{k}\left(V_{k}\left(t\right)-V_{0,k}\right)}-1\right). \end{array}$$

In Equation (4), the subscript *k* denotes either *LA* or *RA*. The volume, end-systolic elastance, and volume-intercept of the end-systolic pressure-volume relationship (ESPVR) of the corresponding atrium are denoted by *V*_*k*_, *E*_*es,k*_, and *V*_0,*k*_, respectively. The parameters *A*_*k*_ and *B*_*k*_ define the atrium curvilinear end-diastolic pressure volume relationship (EDPVR) and the driving function is defined as

(5) $$\begin{array}{l} e(t)=\{\begin{array}{ll} \frac{1}{2}\left(\sin\left[\left(\frac{\pi }{t_{max}}\right)t\;-\frac{\pi }{2}\right]+\;1\right); & \;0<\;t\;\leq \;3t_{max}/2\\ \;\frac{1}{2}\;e^{\frac{-(t-\frac{3t_{max}}{2})}{\tau }}; & t>3t_{max}/2, \end{array}, \end{array}$$

where *t*_*max*_ is the point of maximal chamber elastance and τ is the time constant of relaxation. The time-varying elastance model has been shown to be able to describe atrium contraction well (Hoit et al., 1994).

The relationships between pressures and volumes in the biventricular unit (i.e., LV and RV), pulmonary artery and aorta were computed from their corresponding FE models. These relationships can be expressed as non-closed form functions.

(6a) $$\begin{array}{l} {P_{RV}\left(t\right),P}_{LV}\left(t\right)=\;f^{BV}\left(V_{LV}\left(t\right),\;V_{RV}\left(t\right)\right), \end{array}$$

(6b) $$\begin{array}{l} P_{pa}\left(t\right)=\;f^{PA}\left(V_{pa}\left(t\right)\right), \end{array}$$

(6c) $$\begin{array}{l} P_{sa}\left(t\right)=\;f^{AO}\left(V_{sa}\left(t\right)\right). \end{array}$$

### Finite Element Formulation of the Biventricular Unit

The weak form associated with the biventricular FE model was derived based on minimization of the following Lagrangian functional

(7) $$\begin{array}{l} L_{BV}\left(u_{BV},p_{BV},P_{LV},P_{RV},c_{1,BV},c_{2,BV}\right)\\ =\int_{\Omega_{0,BV}}W_{BV}(u_{BV})dV-\int_{\Omega_{0,BV}}p_{BV}(J_{BV}-1)dV\\ -P_{LV}\left(V_{LV,\text{cav}}(u_{BV})-V_{LV}\right)+P_{RV}\left(V_{RV,\text{cav}}(u_{BV})-V_{RV}\right)\\ -c_{1,BV}\cdot \int_{\Omega_{0,BV}}u_{BV}\;dV-c_{2,BV}\cdot \int_{\Omega_{0,BV}}X_{BV}\times u_{BV}\;dV, \end{array}$$

where Ω_0,*BV*_ is the reference configuration of the biventricular unit, ***u***_*BV*_ is the displacement field, *P*_*LV*_ and *P*_*RV*_ are, respectively, the Lagrange multipliers that constrain the LV cavity volume *V*_*LV, cav*_(***u***_*BV*_) to a prescribed value *V*_*LV*_ and the RV cavity volume *V*_*RV, cav*_(***u***_*BV*_) to a prescribed value *V*_*RV*_ (Pezzuto and Ambrosi, 2014). We note that *V*_*LV*_ and *V*_*RV*_ are prescribed from the closed-loop circulatory model in Equation (6). The Lagrange multiplier *p*_*BV*_ was used to enforce incompressibility of the tissue (i.e., Jacobian of the deformation gradient tensor *J* = 1). The vectors ***c***_1,*BV*_ and ***c***_2,*BV*_ are Lagrange multipliers applied to constrain, respectively, the rigid body translation (i.e., zero mean translation) and rotation (i.e., zero mean rotation) (Pezzuto et al., 2014). In Equation (7), ***X***_*BV*_ denotes a material point in Ω_0,*BV*_ and *W*_*BV*_ is the strain energy function of the myocardial tissue. The cavity volume of the LV and RV were obtained from the displacement field by using the following functional relationship (*k* = *LV* or *RV*)

(8) $$\begin{array}{l} V_{k,cav}(u_{BV})=\;\int_{\Omega_{inner,k}}dV_{k}=-\frac{1}{3}\;\int_{\Gamma_{inner,k}}x_{BV}.\;n\;da_{k}\;, \end{array}$$

where Ω_*inner,k*_ is the volume enclosed by the inner surface Γ_*inner,k*_ of the LV or RV, and ***n*** denotes the outward unit normal vector of those surfaces. Taking the first variation of the Lagrangian functional given in Equation (7) leads to

(9) $$\begin{array}{l} \delta L_{BV}=\int_{\Omega_{0,\;BV}}(P_{BV}-p_{BV}F_{BV}{\;}^{-\text{T}}):\nabla \delta u_{BV}\;dV\\ -\int_{\Omega_{0,\;BV}}\delta p_{BV}(J-1)dV-(P_{LV}\\ +P_{RV})-\int_{\Omega_{0,\;BV}}cof(F_{BV}):\nabla \delta u_{BV}\;dV-\delta P_{LV}(V_{LV,\text{ cav}}(u_{BV})\\ -V_{LV})-\delta P_{RV}(V_{RV,\text{ cav}}(u_{BV})-V_{RV})\\ -\delta c_{1,\;BV}\cdot \;\int_{\Omega_{0,\;BV}}u_{BV}\;dV-\delta c_{2,\;BV}\cdot \;\int_{\Omega_{0,\;BV}}X_{BV}\times u_{BV}\;dV\\ -c_{1,\;BV}\cdot \;\int_{\Omega_{0,\;BV}}\delta u_{BV}\;dV-c_{2,\;BV}\cdot \;\int_{\Omega_{0,\;BV}}X_{BV}\times \delta u_{BV}\;dV. \end{array}$$

In Equation (9), ***P***_*BV*_ is the first Piola Kirchhoff stress tensor and ***F***_*BV*_ is the deformation gradient tensor. The variations of the displacement field, Lagrange multiplier for enforcing incompressibility and volume constraint, zero mean translation, and rotation are denoted by δ***u***_*BV*_, δ*p*_*BV*_, δ*P*_*LV*, cav_, δ*P*_*RV*,cav_, δ***c***_1,*BV*_, and δ***c***_2,*BV*_, respectively. Together with the constraint that the basal deformation at *z* = 0 is in-plane in the biventricular unit, the solution of the Euler-Lagrange problem was obtained by finding $u_{BV}{\in H}^{1}\left(\Omega_{0}\right)$, $p_{BV}\in L^{2}\left(\Omega_{0}\right)$, $P_{LV,\;\text{cav}}\in \;\mathbb{R},P_{RV,\;\text{cav}}\in \;\mathbb{R},c_{1,BV}\in \;{\mathbb{R}}^{3},c_{2,BV}\in \;{\mathbb{R}}^{3}$ that satisfies

(10a) $$\begin{array}{l} {\delta L}_{BV}=0, \end{array}$$

(10b) $$\begin{array}{l} \;u_{BV}\left(x,y,0\right).n{|}_{base}=\;0, \end{array}$$

for all $\delta u_{BV}{\in H}^{1}\left(\Omega_{0}\right),\;\delta p_{BV}\in L^{2}\left(\Omega_{0}\right)$, δ*P*_*LV*, cav_∈ ℝ, δ*P*_*RV*, cav_∈ ℝ, ${\delta c}_{1,\;BV}\in \;{\mathbb{R}}^{3}$, $\delta c_{2,\;BV}\in \;{\mathbb{R}}^{3}.$ The solution of Equation (10) gives the relationship between *P*_*RV*_, *P_LV_*, *V*_*RV*_, *V_LV_* in Equation (6).

### Mechanical Behavior of the Cardiac Tissue

Mechanical behavior of the myocardial tissue was described by an active stress formulation in which the first Piola-Kirchhoff stress tensor ***P***_*BV*_ in Equation (9) was additively decomposed into a passive and an active component, i.e.,

(11) $$\begin{array}{l} P_{BV}=P_{BV,\;p}+P_{BV,\;a\;}e_{f}⊗e_{f_{0}}. \end{array}$$

In Equation (11), ***P***_*BV, p*_ is the passive stress tensor, *P*_*BV, a*_ is the magnitude of the active stress, whereas ***e***_***f***_ and ***e***_***f***_**0**__ are the local basis vectors that define the cardiac muscle fiber directions in the current and reference configuration, respectively. The passive stress tensor ***P***_*BV, p*_ is related to the strain energy function *W*_*BV, p*_ and deformation gradient tensor ***F***_*BV*_ by

(12) $$\begin{array}{l} P_{BV,\;p}=\frac{dW_{BV,p}}{dF_{BV}}\;. \end{array}$$

A Fung-type transversely-isotropic hyperelastic strain energy function (Guccione et al., 1991)

(13a) $$\begin{array}{l} W_{BV,\;p}=\frac{1}{2}C_{BV}\left(e^{Q}-1\right), \end{array}$$

with

(13b) $$\begin{array}{l} Q=b_{ff}E_{ff}^{2}+b_{xx}\left(E_{ss}^{2}+E_{nn}^{2}+E_{sn}^{2}+E_{ns}^{2}\right)\\ \;+b_{fx}\left(E_{fn}^{2}+E_{nf}^{2}+E_{fs}^{2}+E_{sf}^{2}\right) \end{array}$$

was prescribed. In Equation (13b), *E*_*ij*_ with (*i, j*) ϵ (*f, s, n*) denote the components of the Green-Lagrange strain tensor $E=\frac{1}{2}(F_{BV}{\;}^{T}F_{BV}-I)$ with *f*, *s, n* denoting the myofiber, sheet and sheet normal directions, respectively. Material parameters of the Fung-type constitutive model are *C*_*BV*_, *b*_*ff*_, *b*_*xx*_, and *b*_*fx*_.

To describe the active stress behavior, a previously developed active contraction model (Kerckhoffs et al., 2003) was used. The magnitude of the active stress *P*_*BV, a*_ was described by

(14) $$\begin{array}{l} P_{BV,\;a\;}=\frac{l_{s}}{l_{s0}}f^{iso}\left(l_{c}\right)f^{twitch}\left({t,l}_{s}\right)\left(l_{s}-l_{c}\right)E_{a}, \end{array}$$

where *l*_*s*_ is the sarcomere length, *l*_*c*_ is the length of the contractile element, *l*_*s*0_ is the sarcomere length in a prescribed reference state (relaxed sarcomere length), and *E*_*a*_ is the stiffness of the serial elastic element. The function $f^{iso}\left(l_{c}\right)$ denotes the dependency of the isometrically developed active stress on *l*_*c*_ and is given by

(15) $$\begin{array}{l} f^{iso}(l_{c})=\{\begin{array}{ll} T_{0}\;{\text{tanh}}^{2}[{\text{a}}_{6}({\text{l}}_{\text{c}}-{\text{a}}_{7})] & when,\;l_{c}<a_{7}\\ 0 & when,\;l_{c}>a_{7} \end{array}, \end{array}$$

where *T*_0_ is a model parameter that scales the active tension. Both *a*_6_ and *a*_7_ are model parameters. The time course of the active tension development is controlled by

(16a) $$\begin{array}{l} f^{twitch}\left(t,l_{s}\right)=\{\begin{array}{ll} \;0 & when,\;t<0\\ \text{tan}{\text{h}}^{2}\;(\frac{t}{t_{r}})\tanh^{2}\;(\frac{t_{max-}t}{t_{d}}) & when,\;0<t<t_{max}\\ \;0 & when,\;t>0\;, \end{array} \end{array}$$

(16b) $$\begin{array}{l} t_{max}=b\left(l_{s}-l_{d}\right). \end{array}$$

In Equation (16), *t*_*r*_ is the activation rise time constant, *t*_*d*_ is the activation decay time constant, *b* relates activation duration *t*_*max*_ to the sarcomere length *l*_*s*_, and *l*_*d*_ is the sarcomere length at the start of the activation time, i.e., when *t*_*max*_ = 0. The time course of the contractile element *l*_*c*_ was expressed by an ordinary differential equation

(17) $$\begin{array}{l} \frac{\partial l_{c}}{\partial t}=\left[E_{a}\left(l_{s}-l_{c}\right)-1\right]v_{0}, \end{array}$$

where *v*_0_ is the unloaded shortening velocity. The sarcomere length *l*_*s*_ was calculated from the myofiber stretch λ and the relaxed sarcomere length *l*_*s*0_ by

(18a) $$\begin{array}{l} \lambda =\sqrt{e_{f_{0}}\cdot F_{BV}{\;}^{T}F_{BV}e_{f_{0}}}\;, \end{array}$$

(18b) $$\begin{array}{l} l_{s}=\;\lambda l_{s0}. \end{array}$$

### Finite Element Formulation of the Arteries

The pulmonary artery and aorta were modeled as 3D membranes. In the formulation that follows, the subscript *k* = *AO* denotes the aorta and *k* = *PA* denotes the pulmonary artery. Similar to that of the biventricular unit, the finite element formulation of these two arteries can be generalized from the minimization of the following Lagrangian functional, described in the following equation

(19) $$\begin{array}{l} L_{k}(u_{k},P_{k,\text{cav}},c_{1,k},c_{2,k})\\ =\int_{\Omega_{0,k}}W_{k}\;(u_{k})dV-P_{k,\text{cav}}(V_{k,\text{cav}}(u_{k})-V_{k})\\ -c_{1,k}\cdot \;\int_{\Omega_{0,k}}u_{k}\;dV-c_{2,k}\cdot \;\int_{\Omega_{0,k}}X_{k}\times u_{k}\;dV, \end{array}$$

where Ω_0,*k*_ is the reference configuration of the arteries, ***u***_*k*_ is the displacement field and *P*_*k*,cav_ is the Lagrange multiplier that constrains the arterial cavity volume *V*_*k*,cav_(***u***_*k*_) to a prescribed value *V*_*k*_. The vectors ***c***_1,*k*_ and ***c***_2,*k*_ are Lagrange multipliers applied to constrain rigid body motions. The inlet and outlets of the arteries were constrained to move only in-plane. Therefore, the solution of the Euler-Lagrange problem was obtained by finding $u_{k}{\in H}^{1}\left(\Omega_{0}\right),\;P_{k,\text{cav}}\in \;\mathbb{R},c_{1,k}\in \;{\mathbb{R}}^{3},c_{2,k}\in \;{\mathbb{R}}^{3}\;$that satisfies

(20a) $$\begin{array}{l} {\delta L}_{k}=0, \end{array}$$

(20b) $$\begin{array}{l} \;u_{k}\left(x,y,0\right).n{|}_{inlet,\;outlets}=0, \end{array}$$

for all $\delta u_{k}{\in H}^{1}\left(\Omega_{0}\right)$, $\delta P_{k,\text{cav}}\in \;\mathbb{R},\delta c_{1,k}\in \;{\mathbb{R}}^{3},\delta c_{2,k}\in \;{\mathbb{R}}^{3}.$ The solution above gives the relationships between *P*_*pa*_, *V*_*pa*_, and *P*_*sa*_, *V_sa_* in Equations (6b) and (6c), respectively.

### Mechanical Behavior of the Vascular Tissue

The mechanical behavior of the arteries were described by the strain energy function *W*_*k*_ in Equation (21), which is given as the sum of the key tissue constituents, namely, elastin-dominated matrix *W*_*k,e*_, collagen fiber families *W*_*k,c,i*_ and vascular smooth muscle cells (SMC) *W*_*k,m*_ (Baek et al., 2007; Zeinali-Davarani et al., 2011), i.e.,

(21) $$\begin{array}{l} W_{k}=W_{k,e}+\overset{4}{\underset{i=1}{\sum }}\;W_{k,c,i}\;+W_{k,m}. \end{array}$$

Strain energy function of the elastin-dominated amorphous matrix in the arteries is given by

(22) $$\begin{array}{l} W_{k,e}=M_{k,e}\left(\frac{C_{k,1}}{2}\right)\;\left(\text{tr}\left(C_{k}\right)-3\right), \end{array}$$

where *M*_*k,e*_ is the mass per unit volume of the elastin in the tissue, *C*_*k*,1_ is a stiffness parameter and, ${\text{C}}_{\text{k}}={\text{F}}_{\text{k}}^{\text{T}}{\text{F}}_{\text{k}}$ is the right Cauchy-Green deformation tensor associated with the arteries.

In the membrane models, four collagen fiber families were considered. The first and second families of collagen fibers (*i* = 1 and 2) were oriented in the longitudinal and circumferential directions, whereas the third and fourth families of collagen fibers (*i* = 3 and 4) were oriented, respectively, at an angle α = 45° and −45° with respect to the longitudinal axis based on a previous study (Zeinali-Davarani et al., 2011). We assumed that the same strain energy function for all the families of collagen fibers is given by

(23) $$\begin{array}{l} W_{k,c,i}\;=M_{k,i}\frac{c_{k,2}}{{4c}_{k,3}}\left\{\exp\left[c_{k,3}{\left({\lambda_{k,i}}^{2}-1\right)}^{2}\right]-1\right\}. \end{array}$$

In Equation (23), *M*_*k,i*_ is the mass per unit volume of *i*th family of collagen fibers, λ_*k,i*_ is the corresponding stretch of those fibers, and both *c*_*k*,2_ and *c*_*k*,3_ are the material parameters that govern the collagen stiffness. The stretch in the *i*th family of collagen fibers was defined by $\lambda_{k,i}\;=\sqrt{e_{k,i0}\cdot C_{k}e_{k,i0}}$ where ***e***_*k,i*0_ is the local unit vector that defines the corresponding fiber orientation.

Strain energy function of the SMC *W*_*k,m*_ is given by

(24) $$\begin{array}{l} W_{k,m}=M_{k,m}\frac{c_{k,4}}{{4c}_{k,5}}\left\{\exp\left[c_{k,5}{\left({{\text{λ}}_{k,m}}^{2}-1\right)}^{2}\right]-1\right\}. \end{array}$$

Here, *M*_*k,m*_ is the mass per unit volume of the SMC in the tissue, λ_*k,m*_ is the stretch of the SMC, whereas *c*_*k*,4_ and *c*_*k*,5_ are the stiffness parameters. The SMC was assumed to be aligned in the circumferential direction. Mass per unit volume for the different constituents were calculated using following relations

(25a) $$\begin{array}{l} M_{k,e}=\phi_{k,e}\rho , \end{array}$$

(25b) $$\begin{array}{l} M_{k,m}=\phi_{k,m}\rho , \end{array}$$

(25c) $$\begin{array}{l} M_{k,i}=\phi_{k,i}\left(1-\phi_{k,e}-\phi_{k,m}\right)\rho , \end{array}$$

where ϕ_*k,e*_, ϕ_*k,m*_, ϕ_*k,i*_ denote the mass fraction for elastin, SMC and *i*th family of collagen fibers, respectively. Twenty percent of the total collagen mass is assumed to be equally distributed in the longitudinal and circumferential fiber families and the remaining 80% was distributed equally to α = 45° and −45° fiber directions. Constitutive parameters, mass fraction of each constituent and other parameters of the pulmonary artery and aorta membrane models are listed in Table 2.

**Table 2:** Model parameters for FE models for the baseline case.

**Biventricular FE model**	
Passive material model	*C*_*LV*_ = 280 Pa, *C*_*RV*_ = 170 Pa
Active contraction model	*T*_0,*LV*_ = 2000 kPa, *T*_0,*RV*_ = 1800 kPa, *t*_*r*_= 280 ms, *t*_*d*_ = 80 ms, *b* = 0.17 ms.μm^−1^
Circulatory model	*C*_*sv*_ = 0.02 Pa•ml, *C*_*pv*_ = 0.09 Pa•ml, *R*_*sa*_ = 125 kPa•ms•ml^−1^, *R*_*pa*_ = 75 kPa•ms•ml^−1^, *R*_*sv*_ = *R*_*pv*_ = 2 kPa•ms•ml^−1^, *R*_*av*_ = 3.2 kPa•ms•ml^−1^, *R*_*mv*_ = 0.9 kPa•ms•ml^−1^, *R*_*tv*_ = 0.4 kPa•ms•ml^−1^, *R*_*pvv*_ = 2 kPa•ms•ml^−1^, *V*_*sv*,0_ = 3570 ml, *V*_*pv*,0_ = 485 ml
Time varying elastance model of LA and RA	*E*_*es*_ = 60 Pa/ml, *V*_0_ = 10 ml, *t*_*max*_ = 135 ms, τ = 50 ms, *A* = 58.67 Pa, *B* = 0.049 ml^−1^
**Aorta FE model**	
Elastin	*c*_*AO*,1_ = 120 kPa, ϕ_*AO,e*_ = 0.35
Collagen families	*c*_*AO*,2_ = 0.2 kPa, *c*_*AO*,3_ = 8.0, ϕ_*AO,c*_ = 0.20 (ϕ_*AO*,1_ = 0.1ϕ_*AO,c*_, ϕ_*AO*,2_ = 0.1ϕ_*AO,c*_, ϕ_*AO*,3_ = 0.4ϕ_*AO,c*_, ϕ_*AO*,4_ = 0.4ϕ_*AO,c*_)
SMC	*c*_*AO*,4_ = 0.08 kPa, *c*_*AO*,5_ = 3.5, ϕ_*AO,m*_ = 0.45
**Pulmonary artery FE model**	
Elastin	*c*_*PA*,1_ = 45 kPa, ϕ_*PA,e*_ = 0.35
Collagen families	*c*_*PA*,2_ = 100.0 kPa, *c*_*PA*,3_ = 3.0, ϕ_*PA,c*_ = 0.42 (ϕ_*PA*,1_ = 0.1ϕ_*PA,c*_, ϕ_*PA*,2_ = 0.1ϕ_*PA,c*_, ϕ_*PA*,3_ = 0.4ϕ_*PA,c*_, ϕ_*PA*,4_ = 0.4ϕ_*PA,c*_)
SMC	*c*_*PA*,4_ = 5 kPa, *c*_*PA*,5_ = 3.5, ϕ_*PA,m*_ = 0.23

### Solution Algorithm

An explicit time integration scheme was used to solve the ODEs in Equation (1). Specifically, compartment volumes (*V*_*LV*_, *V*_*sa*_, *V*_*sv*_, *V*_*RA*_, *V*_*RV*_, *V*_*pa*_, *V*_*pv*_, *V*_*LA*_) at each time *t*_*i*_ were determined from their respective values and the segmental flow rates (*q*_*mv*_, *q*_*av*_, *q*_*sa*_, *q*_*sv*_, *q*_*tv*_, *q*_*pvv*_, *q*_*pa*_, *q*_*pv*_) at previous time *t*_*i*−1_ in Equation (2). The computed compartment volumes at *t*_*i*_ were used to update the corresponding pressures (*P*_*LA*_, *P*_*RA*_, *P*_*LV*_, *P*_*RV*_, *P*_*sa*_, *P*_*pa*_, *P*_*sv*_, *P*_*pv*_). Pressures in the atrium (*P*_*LA*_, *P*_*RA*_) and veins (*P*_*sv*_, *P*_*pv*_) were computed from Equations (4) and (3), respectively. On the other hand, pressures in the LV (*P*_*LV*_), RV (*P*_*RV*_), were computed from the FE solutions of Equation (10) for the biventricular unit with the volumes (*V*_*LV*_, *V*_*RV*_) at time *t*_*i*_ as input. Similarly, pressures in the aorta (*P*_*sa*_) and pulmonary artery (*P*_*pa*_) were computed from the FE solutions of Equation (20) with their corresponding volumes (*V*_*sa*_, *V*_*pa*_) at time *t*_*i*_. We note here that (*P*_*LV*_, *P*_*RV*_, *P*_*sa*_, *P*_*pa*_) are scalar Lagrange multipliers in the FE formulation for constraining the cavity volumes to the prescribed values (*V*_*LV*_, *V*_*RV*_, *V*_*sa*_, *V*_*pa*_). The computed pressures at time *t*_*i*_ were then used to update the segmental flow rates in Equation (2) that will be used to compute the compartment volumes at time *t*_*i*+1_ in the next iteration.

### Model Parameterization and Simulation

The biventricular FE model was divided into three material regions, namely the LV free wall (LVFW), the septum, and the RV free wall (RVFW). Similar to a previous study (Finsberg et al., 2018), passive stiffness *C* and contractility *T*_0_ were prescribed to be the same values in the LVFW and septum (denoted as *C*_*LV*_ and *T*_0,*LV*_) and had different values in the RVFW (denoted as *C*_*RV*_ and *T*_0,*RV*_). In the baseline case, model parameters were adjusted to fit the clinically measured LV and RV PV loops, volume and pressure waveforms throughout the cardiac cycle. Specifically, the LV and RV end diastolic pressures were matched by adjusting the passive stiffness parameters *C*_*LV*_ and *C*_*RV*_. Stroke volume (SV) of the LV and RV were matched by adjusting the regional contractility parameters (i.e., *T*_0,*LV*_*, T*_0,*RV*_). While other model parameters can also affect the SV (e.g., peripheral resistances *R*_*sa*_ *and R*_*pa*_ of the systemic and pulmonary circulations as well as preload), the parameters *T*_0,*LV*_ and *T*_0,*RV*_, which scale the active stress generated by the myofiber, have a larger effect on the LV and RV SV, respectively. On the other hand, the contraction model parameters *t*_*r*_, *t*_*d*_ and *b* were adjusted to match the time course of the volume and pressure waveforms measured in the LV and RV. Parameters *t*_*r*_ and *t*_*d*_ were adjusted to match the time to peak tension and *b* was adjusted to achieve the desirable relaxation of the myofibers. Circulatory model parameters (resistances and compliances) were also adjusted to match the systolic pressure (afterload), preload and systemic and pulmonary vein pressures. Aortic and PA peripheral resistances (*R*_*sa*_, *R*_*pa*_) were calibrated to match the systolic pressures of LV and RV. The parameters related to LA and RA time-varying elastance models were prescribed based on a previous study (Shavik et al., 2019). Parameters related to the aorta and PA constitutive models (that alter the vessel's compliance) were adjusted to match the measured pressure waveforms, and the diameters estimated from the PC-MRI. All the model parameters for the biventricular, aorta and PA FE models are listed in Table 2.

The multiscale modeling framework was implemented using FEniCS (Alnæs et al., 2015). The biventricular unit was meshed with ~7,700 tetrahedral elements based on a previous study (Finsberg et al., 2018) showing that local fiber stress and global features related to cardiac contraction are not sensitive to mesh resolution beyond ~4,000 elements. Furthermore, the aorta and pulmonary arteries FE models contain ~8,000 triangular elements based on previous study (Zeinali-Davarani et al., 2011) that used ~1,500 elements. Steady state PV loop was established by running the simulation over several cardiac cycles until cycle-to-cycle periodicity was achieved. The prescribed cardiac cycle time (690 ms) was derived from the heart rate (87 bpm) measured during the PC-MRI acquisition.

Since it is known that key features of the progression of PAH include stiffening of main PA, reduced RV contraction, and increased distal resistance of PA (Fan et al., 1997; Shimoda and Laurie, 2013), we used our calibrated model to investigate how these changes affect the cardiopulmonary circulation. Specifically, a sensitivity analysis on the parameters associated with PAH progression was performed by simulating the following cases: (1) a 67% increase in PA collagen mass fraction ϕ_*PA,c*_, (2) a 50% decrease in RV contractility *T*_0,*RV*_, and (3) a 50% increase in the pulmonary arterial resistance *R*_*pa*_.

## Results

### Comparison Between Simulated Results and Clinical Measurements

**Figure 3:**
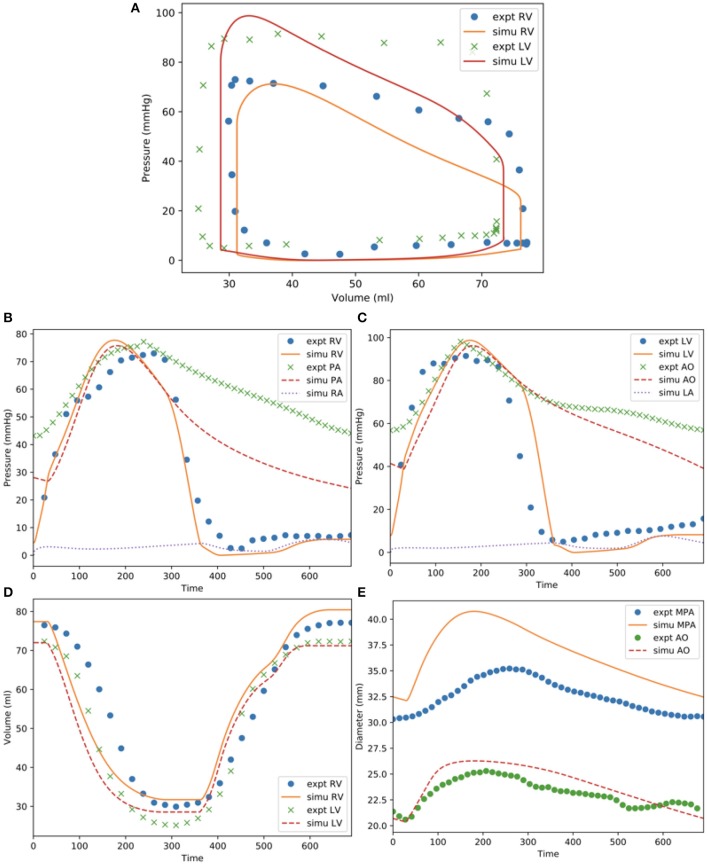
Measurements and model predictions for the baseline case. **(A)** LV and RV PV loops; **(B)** pressure waveforms of pulmonary circulation; **(C)** pressure waveforms of systemic circulations; **(D)** LV and RV volume waveforms; **(E)** MPA and AO diameter waveforms.

**Figure 4:**
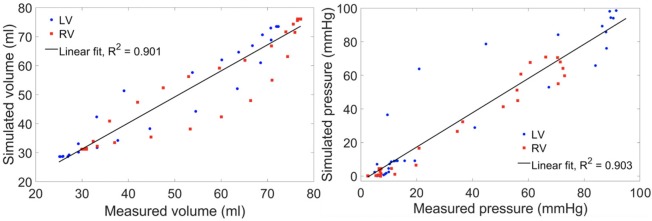
Scatter plot of the simulated vs. measured volume **(left)** and pressure **(right)** at all cardiac time points of the baseline case with a linear fit showing the zero-error reference.

Model predictions of the LV and RV PV loops, volume waveforms, and pressure waveforms in the baseline case matched reasonably well with the clinically measured PAH patient data described in Section Data Acquisition (Figure 3). Good overall fitting was obtained for the volume and pressure in both the LV and RV with the coefficients of determination R^2^ value of 0.901 and 0.903, respectively (Figure 4). Pressure waveforms in the pulmonary and systemic circulations predicted by the model also agree, in general, reasonably well with the measurements, except for the diastolic pressure. The model predicted smaller diastolic pressure in the aorta (by ~17 mmHg) and PA (by ~15 mmHg) when compared to the measurements (Figure 3B). The simulated ascending AO and PA diameter waveforms compared well with the clinical measurements of the dynamic cross-sectional area from the PC-MRI (Figure 3C). Specifically, the simulated and clinically measured diameter waveforms in the ascending AO are in good agreement (max. abs difference ~10%) while the model predicted a larger change of the diameter compared to the measurements for the MPA (max. abs difference ~28%).

### Effects of the Changes in Vascular Microstructure on Cardiac Function

**Figure 5:**
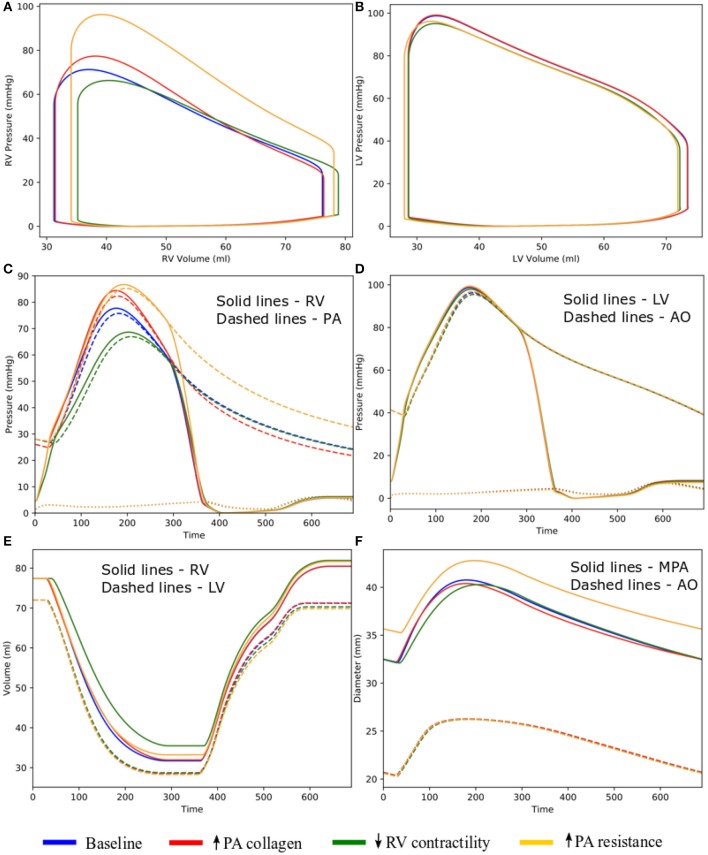
Hemodynamic comparison between the different simulations. **(A)** RV and **(B)** LV PV loops; **(C)** Pulmonary and **(D)** systemic circulation pressure waveforms; **(E)** LV and RV volume waveforms; and **(F)** MPA and AO diameter waveforms.

**Figure 6:**
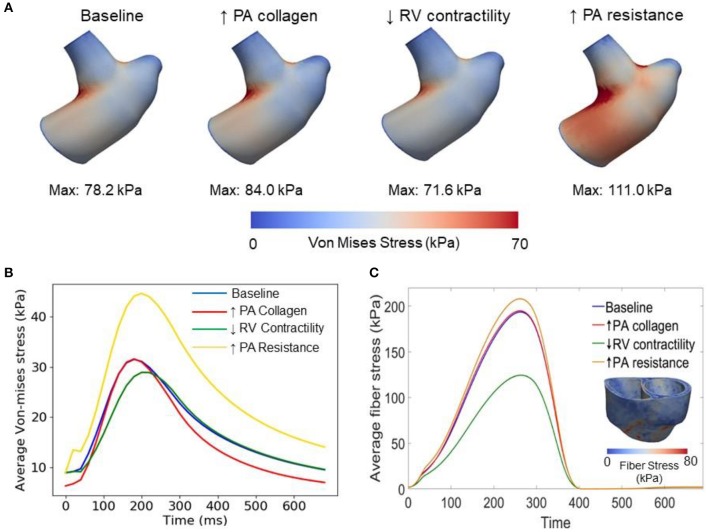
Comparison of wall stresses in the different simulations. **(A)** Von-mises stress map of the pulmonary artery FE model. **(B)** Average von-mises wall stress waveforms in the pulmonary artery FE model. **(C)** Average fiber stress waveforms in the biventricular FE model and fiber stress map of baseline model.

Changing the mass fractions of the constituents in the PA wall led to changes in its function, which in turn affects the RV function. Specifically, increasing the mass fraction ϕ_*PA,c*_ of the collagen of PA wall by 67% (from 0.42 to 0.70) with a corresponding decrease in the mass fraction of the elastin (from 0.35 to 0.15) and SMC (from 0.23 to 0.15) produced an increase in the PA pressure of 10% (from 71 to 78 mmHg). The RV systolic pressure also increased by 11% (from 68 to 76 mmHg) correspondingly (Figure 5A). Because of the more exponential mechanical response of the PA with higher collagen fraction, the PA pressure also decayed more rapidly during the diastolic phase resulting in an increased pulse pressure (from 45 mmHg baseline to 55 mmHg) (Figure 5C). The LV and RV SV and EF remained relatively unchanged (Figures 5A,B). In the aorta, systolic, diastolic, and pulse pressures did not change significantly from the baseline case (Figure 5D). The change in PA diameter was slightly reduced when compared to baseline (Figure 5F) as the vessel becomes stiffer with higher collagen mass fraction. Spatially averaged RV fiber stress did not change when compared to the baseline case. Maximum arterial wall stress located at the bifurcation increased (~7.4%) but the spatially averaged wall stress did not change significantly from baseline (Figure 6).

### Effects of the Change in RV Contractility on Vasculature

Decreasing the RV contractility *T*_0,*RV*_ by 50% (from 1,800 kPa baseline to 900 kPa) reduced the RV EF by 5% (from 58 to 53%) (Figure 5A). Due to less contractile force being generated by the RV, both RV and PA peak systolic pressure decreased by about 9% (RV: 71 to 65 mmHg; PA: 68 to 62 mmHg) (Figure 5C). In addition, the LV EF as well as peak systolic pressure in both the LV and aorta were slightly decreased compared to the baseline (Figures 5B,D,E). Because of the reduced pressure, PA diameter was slightly reduced during systole when compared to baseline (Figure 5F). Average RV fiber stress also decreased by 37% (from 195 to 124 kPa) compared to baseline. Both maximum arterial and spatially averaged RV wall stress were reduced by about 9% (Figure 6).

### Effects of the Change in PA Resistance

Increasing the pulmonary arterial resistance *R*_*pa*_ by 50% led to an increase in PA pulse pressure by 36% (from 45 to 61 mmHg), which was also accompanied by an increase in PA systolic and diastolic pressure (Figure 5C). The RV peak systolic pressure increased by 34% (from 71 to 95 mmHg) and the RV EF decreased by 2% (from 58 to 56%) (Figure 5A). Due to the higher pressure, the PA diameter waveform shifted upwards and became higher than the baseline throughout the cardiac cycle. Similar to the case with reduced RV contractility, LV EF as well as peak systolic pressure in both the LV and aorta were slightly decreased compared to the baseline (Figures 5B,D,E). A 7% (195 to 208 kPa) increase in average RV fiber stress as well as a 41% increase in maximum arterial wall stress were also found in the PA (Figure 6).

## Discussion

In order to characterize the intricate progression of PAH, we developed the first closed-loop multiscale modeling framework (consisting of image-based FE models of the left and right ventricles, large pulmonary arteries, and aorta) that captures detailed bi-directional ventricular-arterial interactions. We have shown that our proposed model describes the cardiopulmonary circulation reasonably well by reproducing patient-specific measurements of (1) LV and RV PV loops, (2) LV and RV volume and pressure waveforms, and (3) aorta and PA pressure and diameter waveforms of a PAH patient.

This framework extends our previously developed hybrid lumped-FE model of the systemic circulation (Shavik et al., 2018) by including the RV, large pulmonary arteries and the pulmonary micro-circulation (represented with a lumped model). Previous modeling frameworks have coupled a FE biventricular model with a lumped representation of the pulmonary circulation (Kerckhoffs et al., 2007; Xi et al., 2016) but not with FE model of the large pulmonary arteries. The ability to couple a FE model of the large arteries and both ventricles in this framework enables us to investigate PAH progression reflected in the large pulmonary arteries and the RV. Specifically, the framework allows us to alter the microstructural, geometrical and mechanical behaviors of the pulmonary arteries and characterize how these changes affect the RV, and vice versa. Implementing 3D FE models of the arteries in the framework also allow us to capture non-homogeneous stress distribution in the vessels (e.g., high stress concentration at the bifurcation of the pulmonary artery in Figure 6) which would not be possible using lumped-parameter models. Using the calibrated framework, we have created three cases to simulate progressive pathological changes associated with PAH in the (1) large pulmonary arteries (increase in collagen mass and degradation of elastin) (Wang et al., 2013), (2) RV (decrease in contractility due to right ventricular failure) (Naeije and Manes, 2014), and (3) pulmonary microcirculation (increase in resistance due to remodeling) (Kobs et al., 2005).

Increasing the collagen mass in the elastic proximal pulmonary arteries increased PA pulse pressure from baseline. This behavior is due to the stiffening of the PA, which results from a more exponential stress-strain behavior associated with the higher concentration of collagen fibers. This result is consistent with animal experiments where an increase in PA pulse pressure has been associated with an increase in collagen mass in PAH (Wang et al., 2017). Furthermore, the connection between pulse pressure and changes in collagen can also be found in the aorta during aging, where a loss of elastin (which results in a more collagen-dominated extracellular matrix) produces an increase in systemic pulse pressure (Safar et al., 2003). A decrease in PA compliance that is caused by an increase in collagen mass produced an increase in RV afterload as reflected by an increase in RV systolic pressure in our model, consistent with previous studies (Mahapatra et al., 2006; Gan et al., 2007). Consistent with our previous study (Shavik et al., 2018), the more pulsatile PA waveform can also be observed in the ejection phase of the RV PV loop, where the pressure-volume curve became steeper toward end-of-systole (Figure 5A). Our model did not predict a significant reduction in the SV, which could be attributed to a high RV end-systolic elastance in the model. We note that a high RV end-systolic elastance has also been associated with PAH (Vélez-Rendón et al., 2018), especially during the compensatory phase.

Decreasing RV contractility (by 50%) in the model, which reflects the transition to decompensated heart failure, produced an expected decrease in EF and peak systolic pressure that results in a substantial decrease in myofiber stress in the RV. Reducing the RV contractility also reduces the PA peak and pulse pressures, only decreasing the arterial wall stress in the PA slightly. Based on consensus that arterial wall stress is the driver for vascular remodeling (Humphrey, 2008), this result suggests that remodeling in the large pulmonary arteries may attenuate the transition to the decompensated phase. This result also suggests that negative inotropic agent targeted at the RV may help attenuate remodeling in the PA vasculature.

Lastly, increasing the distal pulmonary arterial resistance, which reflects remodeling of the distal vessels, increased pressures in the proximal PA and RV. A 50% increase in the distal pulmonary arterial resistance (equivalent to a ~10% reduction of the vascular lumen diameter based on Poiseuille's law) causes ejection to start at a higher pressure and the EF to be slightly reduced in the RV. These results are broadly consistent with the effects on the RV measured in patients under acute hypoxia (Akgül et al., 2007), which shows an increase in both end-systolic and end-diastolic volume and a slight (but not significant) decrease in EF. The same increase in resistance also produced a significantly higher increase in the systolic PA pressure than the simulation with a 67% increase in collagen mass in the proximal pulmonary arteries. These results suggest that remodeling in the microcirculation contributes more to changes in the pulmonary pressure than remodeling in the proximal pulmonary arteries, suggesting that PAH is primarily driven by distal arterial remodeling. In summary, we have shown that isolated changes in both the arteries and ventricles as predicted by our modeling framework lead to expected effects in the cardiopulmonary circulation. This confirms that the modeling framework can capture bi-directional ventricular-arterial interactions, which can be used to further our understanding of PAH progression.

## Model Limitations

Though our modeling framework is able to predict behaviors that are consistent with the measurements there are, however, some limitations associated with it. First, the local myofiber orientation was varied transmurally from 60° in the endocardium to −60° at the epicardium using a “rule based” method. Thus, we did not take into account any changes in myofiber orientation during RV remodeling (Hill et al., 2014) that may occur in PAH. Second, we have assumed a uniform wall thickness and homogeneous material properties for both aorta and PA in our model. We believe that this assumption contributes to the mismatch in the MPA diameter waveforms. Third, we have assumed that FE models of the pulmonary arteries and aorta account for the compliance of the entire pulmonary and systemic arterial system, respectively. This is a limitation because the FE models are associated with only a segment of their corresponding arterial systems. We show in a preliminary study (see Appendix) that the addition of a lumped-parameter compliance to the modeling framework can be used to provide a better match of pulmonary artery pressure and diameter waveforms, as well as the pressure-volume loops. Fourth, we have neglected the dynamic behavior of the fluid and its interaction with the vessel walls and the spatial variation of pressure waveform along the aortic and pulmonary tree and shear stress on the luminal surface of the vessels. We note, however, that the computed shear stress (~Pa) is several order of magnitude smaller than the normal traction force (pressure) on the surface of the vessel (~kPa) and variation of peak pressure within the vessel is <10%. For these reasons, the omission of shear traction should not affect the computed arterial stresses. Last, the modeling framework was calibrated using data acquired from one PAH patient. Caution must be exercised in extrapolating results to the general population of pediatric PAH patients.

## Data Availability Statement

The datasets generated for this study are available on request to the corresponding author.

## Ethics Statement

This study was approved by the University of Michigan Board of Review (HUM00117706). Written informed consent to participate in this study was provided by the participants' legal guardian/next of kin.

## Author Contributions

SS, SB, and LL developed the theoretical formulation and computational framework of the model. SS and CT-B carried out the simulations for different cases and prepared the results. CT-B and CF acquired the clinical data. LL, SB, and CF planned and supervised the work. All authors helped in interpretation of the results and contributed to the final manuscript.

### Conflict of Interest

The authors declare that the research was conducted in the absence of any commercial or financial relationships that could be construed as a potential conflict of interest.

## Abbreviations

AO: Aorta
EDPVR: End-diastolic Pressure Volume Relationship
EF: Ejection Fraction
ESPVR: End-systolic Pressure Volume Relationship
FE: Finite Element
HR: Heart Rate
LA: Left Atrium
LPA: Left Pulmonary Artery
LV: Left Ventricle
LVEDV: Left Ventricular End-diastolic Volume
LVEF: Left Ventricular Ejection Fraction
LVESV: Left Ventricular End-systolic Volume
LVFW: Left Ventricular Free Wall
MAP: Mean Aortic Pressure
MPA: Main Pulmonary Artery
mPAP: Mean Pulmonary Arterial Pressure
MR: Magnetic Resonance
PA: Pulmonary Artery
PAH: Pulmonary Arterial Hypertension
PC-MRI: Phase-Contrast Magnetic Resonance Image
PCWP: Pulmonary Capillary Wedge Pressure
PV: Pressure-Volume
pv: Pulmonary Veins
PVR: Pulmonary Vascular Resistance
RA: Right Atrium
RPA: Right Pulmonary Artery
RV: Right Ventricle
RVEDV: Right Ventricular End-diastolic Volume
RVEF: Right Ventricular Ejection Fraction
RVESV: Right Ventricular End-systolic Volume
RVFW: Right Ventricular Free Wall
sa: Systemic Arteries
SMC: Smooth Muscle Cells
sv: Systemic Veins
WU: Wood Unit.
