# Supplementary material for: Multiscale Modeling Framework of Ventricular-Arterial Bi-directional Interactions in the Cardiopulmonary Circulation
Source: Front Physiol. 2020 Jan 31;11:2. doi: 10.3389/fphys.2020.00002 (PMC7025512; doi:10.3389/fphys.2020.00002)
Supplement: Supplementary file 1 [file Data_Sheet_1.docx]

**Appendix**

We performed an additional study to determine if the addition of compliance (captured by an electrical analog of a capacitor) in parallel to the pulmonary arterial system (**Fig. S1a**) can match clinically-measured pressure and diameter waveforms more accurately. To do so, Eq. (1f) was modified as follows:

$\frac{dV_{pa}(t)}{dt}=q_{pvv}\left( t \right)-q_{pa}\left( t \right)-q_{pa2}\left( t \right)$ (A1a)

$q_{pa2}=C_{pa}\frac{{dP}_{pa}}{dt}$ (A1b)

with$C_{pa}=0.0025 Pa\bullet ml$ as well as increasing the stiffness of the pulmonary arteries with the parameters $c_{PA,1}$ = 90kPa, $c_{PA,2}=200\mathrm{kPa}, c_{PA,4} = 10 kPa.$ This addition of compliance led to a better match of the pulmonary artery pressure and diameter waveforms, as well as the pressure-volume loops with the experimental data (**Fig. S1b - d**).


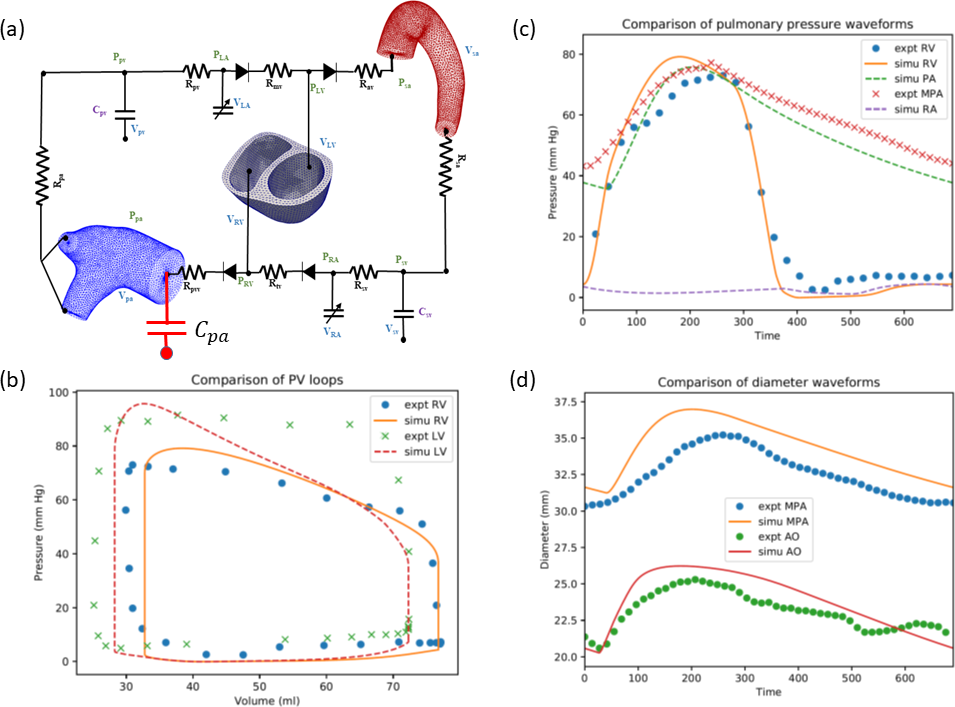


**Figure S1 (a):** Modified framework with an additional compliance (in red) in the pulmonary circulation**.** Comparison of **(b):** pressure-volume loops**, (c):** pressure waveforms of the pulmonary circulation and **(d)** pressure-diameter waveform**.**
